# Detection of Bacterial Membrane Vesicles by NOD-Like Receptors

**DOI:** 10.3390/ijms22031005

**Published:** 2021-01-20

**Authors:** Ella L. Johnston, Begoña Heras, Thomas A. Kufer, Maria Kaparakis-Liaskos

**Affiliations:** 1 Department of Physiology, Anatomy and Microbiology, La Trobe University, Bundoora 3086, Australia; eljohnston@students.latrobe.edu.au; 2 Research Centre for Extracellular Vesicles, School of Molecular Sciences, La Trobe University, Bundoora 3086, Australia; 3 Department of Biochemistry and Genetics, La Trobe Institute for Molecular Sciences, La Trobe University, Bundoora 3086, Australia; b.heras@latrobe.edu.au; 4 Department of Immunology, Institute for Nutritional Medicine, University of Hohenheim, 70593 Stuttgart, Germany; thomas.kufer@uni-hohenheim.de

**Keywords:** bacterial membrane vesicles (BMVs), outer membrane vesicles (OMVs), membrane vesicles (MVs), NOD-like receptors (NLRs), NODs, NOD1, NOD2, bacterial pathogenesis, inflammasome, NLRP3

## Abstract

Bacterial membrane vesicles (BMVs) are nanoparticles produced by both Gram-negative and Gram-positive bacteria that can function to modulate immunity in the host. Both outer membrane vesicles (OMVs) and membrane vesicles (MVs), which are released by Gram-negative and Gram-positive bacteria, respectively, contain cargo derived from their parent bacterium, including immune stimulating molecules such as proteins, lipids and nucleic acids. Of these, peptidoglycan (PG) and lipopolysaccharide (LPS) are able to activate host innate immune pattern recognition receptors (PRRs), known as NOD-like receptors (NLRs), such as nucleotide-binding oligomerisation domain-containing protein (NOD) 1, NOD2 and NLRP3. NLR activation is a key driver of inflammation in the host, and BMVs derived from both pathogenic and commensal bacteria have been shown to package PG and LPS in order to modulate the host immune response using NLR-dependent mechanisms. Here, we discuss the packaging of immunostimulatory cargo within OMVs and MVs, their detection by NLRs and the cytokines produced by host cells in response to their detection. Additionally, commensal derived BMVs are thought to shape immunity and contribute to homeostasis in the gut, therefore we also highlight the interactions of commensal derived BMVs with NLRs and their roles in limiting inflammatory diseases.

## 1. Introduction

**Figure 1:**
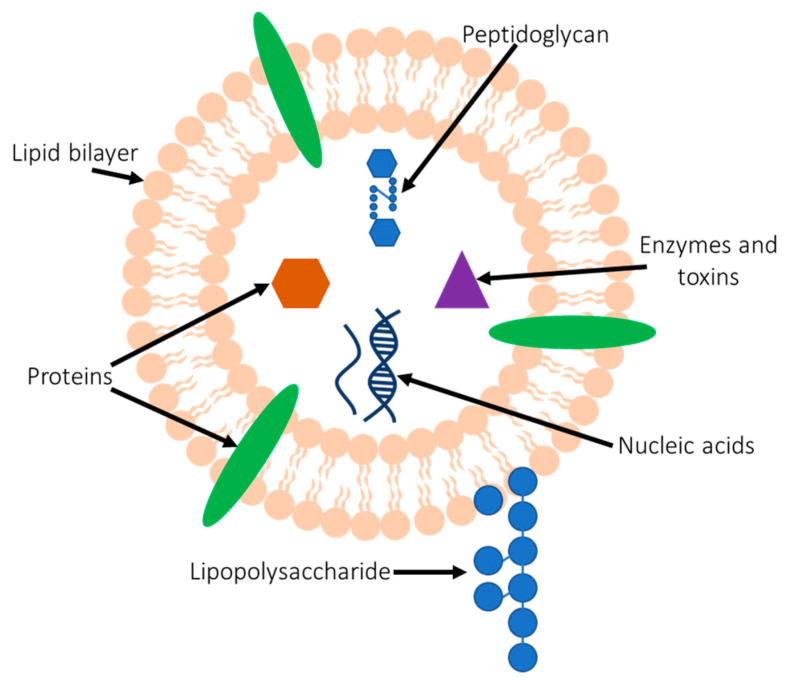
Schematic of a bacterial membrane vesicle. Bacterial membrane vesicles (BMVs), which includes outer membrane vesicles (OMVs) produced by Gram-negative bacteria and membrane vesicles (MVs) produced by Gram-positive bacteria, are nanoparticles produced by all bacteria as part of their normal growth. BMVs contain a range of immunostimulatory microbe-associated molecular patterns (MAMPs) from their parent bacterium, such as peptidoglycan, lipopolysaccharide, DNA and RNA, in addition to bacterial specific virulence determinants such as toxins and enzymes that can mediate the induction of an inflammatory response and promote disease in the host.

Bacteria have a range of mechanisms they use to interact with the host in order to ultimately modulate immunity or to promote disease. A common mechanism used by bacteria to deliver biological cargo to the host and which results in the activation of innate immune receptors, such as Toll-like receptors (TLRs) and NOD-like receptors (NLRs), involves bacterial membrane vesicles (BMVs) [1,2]. BMVs are naturally produced nanostructures, ranging from approximately 20–300 nm in size, that are released by all bacteria as part of their normal growth [3]. Initially believed to be artefacts of bacterial growth, it is now well established that BMVs are a conserved secretion mechanism used by bacteria to package proteins, nucleic acids and virulence determinants that can be delivered to host cells to modulate cellular functions and the development of an immune response (Figure 1) [1,4]. Both Gram-negative and Gram-positive bacteria produce vesicles, which we collectively refer to as BMVs; however, there are some differences between the type of vesicles they produce and their mechanisms of biogenesis. Specifically, BMVs produced by Gram-negative bacteria which have an outer membrane are called outer membrane vesicles (OMVs), whereas BMVs produced by Gram-positive bacteria are released from the cellular membrane of the bacterium and are referred to as membrane vesicles (MVs).

The contribution of BMVs to mediating pathogenesis and modulating immunity is well established. BMVs have been observed within a range of tissues and biopsies obtained from humans with active bacterial infections, which include gastric tissues, blood and cerebral spinal fluid, indicating their clinical relevance to mediating pathology [5,6,7]. Similarly, pathogen derived BMVs have been demonstrated to contribute to disease. For example, *Escherichia coli* OMVs have been shown to increase the ability of bacteria to invade host epithelial cells and may be a contributing factor to Crohn’s disease and inflammatory bowel disease (IBD) [8]. Furthermore, OMVs produced by the gastric pathogen *Helicobacter pylori* can increase the production of the chemokine CXCL8 (interleukin-8, IL-8) by gastric epithelial cells to promote inflammation in the host [9]. In addition, BMVs containing immunostimulatory cargo are able to interact with surface or cytosolic pattern recognition receptors (PRRs) to modulate immunity. The activation of PRRs by BMVs results in the induction of a proinflammatory response, thus contributing to mediating inflammation and pathogenesis within the host [2,10,11,12]. Therefore, understanding the mechanisms whereby bacteria package immunostimulatory cargo within BMVs and their ability to activate host PRRs is integral to understanding their roles during infection and host–microbe homeostasis. Here, we discuss the cargo composition of BMVs and the mechanisms whereby they interact with a range of host PRRs, in particular NLRs, to modulate immunity and promote disease in the host. We discuss how OMVs and MVs produced by Gram-negative and Gram-positive bacteria can activate NLRs respectively, and we will collectively refer to them as BMVs.

## 2. BMVs Contain a Range of Immunostimulatory Cargo

A number of bacterial species have been shown to release BMVs containing immunostimulatory cargo known as microbe-associated molecular patterns (MAMPs), which includes peptidoglycan (PG), lipopolysaccharide (LPS), DNA and RNA (Figure 1) [10,13,14,15,16,17]. Furthermore, numerous studies have reported the ability of BMVs containing MAMPs to activate a range of host PRRs. For example, OMVs produced by *Pseudomonas aeruginosa*, *Bordetella pertussis* and *Porphyromonas gingivalis* were found to contain the endotoxin LPS [13,16,18]. Specifically, the presence of LPS associated with OMVs produced by Gram-negative bacteria could activate TLR4 on the surface of host cells as well as inflammasomes within the host cell cytosol, showing that OMV-associated LPS is biologically active and immunostimulatory in a range of epithelial and innate immune cells, including dendritic cells [19,20,21,22,23]. In addition, OMVs and MVs produced by Gram-negative and Gram-positive bacteria have been shown to activate a range of TLRs including TLR2, TLR4 and TLR9 as a result of their immunostimulatory MAMP cargo. For example, *Acinetobacter baumannii* OMVs have been shown to activate TLR2 and TLR4 to induce inflammation in mice [24], and OMVs produced by enterohaemorrhagic *E. coli* could signal via TLR4 and TLR5 in human epithelial cells, which induced the production of proinflammatory cytokines [25]. OMVs produced by periodontal pathogens are also able signal via TLR2 and TLR4 in addition to activating TLR9 [26]. Furthermore, BMVs have also been shown to indirectly impair TLR signalling. For example, the pathogen *Brucella abortus* produces OMVs which, when preincubated with THP-1 monocytes, can inhibit TLR2, TLR4 and TLR5 responses prior to challenge with TLR ligands, suggesting that OMVs can also have an immunomodulatory role by functioning to suppress TLR-mediated responses [27]. Additionally, OMVs produced by the commensal gut bacterium *Bacteroides fragilis* are suggested to modify the gene expression of TLR2 and TLR4 in epithelial cells, as well as inducing the production of anti-inflammatory cytokines. This suggests that OMVs may be a mechanism used to dampen immune responses to the gut microbiota in order to maintain homeostasis [28]. 

**Figure 2:**
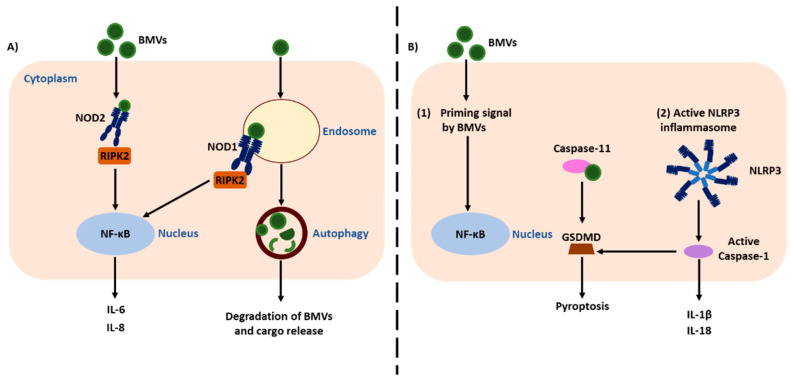
Activation of NLRs by BMVs in host cells. BMVs can enter host cells using a number of mechanisms, whereby access to NOD-like receptors (NLRs) occurs. (**A**) Internalised BMVs interact with NOD1 and NOD2, which can lead to endosomal localisation of NOD1, NF-ĸB activation and the production of proinflammatory cytokines. Furthermore, once intracellular, BMVs can be degraded by the host cellular degradation pathway of autophagy in a NOD1-dependent manner. (**B**) BMVs can also prime host cells for NLRP3 inflammasome activation, which leads to cleavage of GSDMD, pyroptosis and the production of proinflammatory cytokines. BMVs have also been shown to interact with caspase-11, inducing inflammasome activation and the release of proinflammatory cytokines.

Additionally, PG has also been identified as a component of BMVs that is capable of being detected by PRRs. It was initially shown using OMVs from the mucosal pathogens *H. pylori*, *Neisseria gonorrhoeae* and *P. aeruginosa* that OMVs contained PG and that upon entry into host epithelial cells, OMV-associated PG was accessible and detected by the cytoplasmic NLR, nucleotide-binding oligomerisation domain-containing protein 1 (NOD1), resulting in the production of a pro-inflammatory immune response and the generation of an OMV-specific antibody response in vivo (Figure 2A) [10]. This study therefore identified that BMVs are a novel mechanism whereby bacteria, irrespective of their mode of infection, can deliver proinflammatory MAMPs to the intracellular compartment of host cells to activate PRRs and drive an inflammatory response. This finding was further supported by numerous examples showing the ability of OMVs produced by other pathogens to activate both NOD1 and NOD2 responses in host cells [29,30,31,32,33]. Collectively, these studies indicate that the delivery of MAMPs using BMVs produced by Gram-negative and Gram-positive bacteria is an important aspect of PRR and NLR activation in the context of host-microbe interactions. In this review, we will focus on the mechanisms whereby BMVs activate NLR-dependent responses. The mechanisms whereby BMVs activate TLR responses are discussed in detail elsewhere [1,2,12,34].

## 3. The NOD-Like Receptors NOD1 and NOD2 Detect BMVs

NOD-like receptors (NLRs) are a family of intracellular innate PRRs, consisting of 22 NLRs in humans, which contribute to host immunity [35]. Activation of NLRs via the cytosolic recognition of their ligands leads to the activation of nuclear factor “kappa-light-chain-enhancer” of activated B cells (NF-ĸB), mitogen-activated protein kinase (MAPK) signalling and ultimately the production of inflammatory cytokines [36]. The first NLRs that were identified to be responsible for the detection of BMVs and for contributing to the development of BMV-mediated innate and adaptive immune responses were the nucleotide-binding oligomerisation domain-containing proteins 1 and 2 (NOD1 and NOD2, respectively) [10,29]. NOD1 and NOD2 are cytosolic NLRs, which were first identified approximately 20 years ago, and are responsible for the detection of bacterial PG within the host cell cytosol [37,38]. Specifically, NOD1 and NOD2 detect key components of bacterial peptidoglycan being D-glutamyl-meso-diaminopimelic acid (iE-DAP) [39], and muramyl dipeptide (MDP), respectively [40]. NOD1 and NOD2 receptors contain one or two caspase activation and recruitment domains (CARD), respectively, in addition to a nucleotide-binding and oligomerisation domain (NACHT) that enables homodimerisation and a leucine rich repeat domain (LRR) which is responsible for the recognition of PG [37,38]. The detection of PG by NOD1 and NOD2 has been shown to lead to recruitment of the adaptor molecule receptor-interacting serine/threonine kinase 2 (RIPK2) [41,42]. Therefore, upon ligand recognition, NOD1 and NOD2 recruit RIPK2 through a CARD-CARD interaction, whereby RIPK2 acts as a scaffolding protein to provide a platform for downstream signalling events [35,43]. This in turn leads to the activation of NF-ĸB and the production of proinflammatory cytokines [35,37,38,43]. In addition, NOD1 and NOD2 activation can also mediate signalling via the MAPK pathway, whereby kinases such as extracellular signal-regulated kinase (ERK) phosphorylate transcription factors in the nucleus to induce the expression of proinflammatory cytokines [35,43]. 

Despite our understanding that NOD1 and NOD2 detect bacterial PG to mediate an inflammatory response, much about these receptors remains unknown. For example, the complete structures of human NOD1 and NOD2 remain to be elucidated, which has significantly impacted the advancement of this field. However, studies determining a partial NOD1 structure in addition to determining the structure of rabbit NOD2 protein suggest that dimerisation is essential for RIPK2 interaction, and that a number of residues may be important for the localisation of NOD1 and NOD2 to the plasma membrane during activation [44,45]. In addition to the unknown structures of NOD1 and NOD2, their exact intracellular location remains unknown. To date, it is proposed that NOD1 is located in the cytoplasm. However, upon its activation by OMVs in epithelial cells, NOD1 is recruited to the endosomal membrane, whereas NOD1 has also been shown to be recruited to the plasma membrane when activated by bacteria [31,46]. Similarly, NOD2 has been shown to be recruited to endosomes upon activation in response to bacterial infection in macrophages [47]. In addition, recent studies have identified that posttranslational modifications of adaptor proteins can mediate the effects of NOD1 and NOD2 activation. For example, it has been reported that ubiquitination is an important contributor to the regulation of NODs, whereby ubiquitin regulates RIPK2 interactions with NOD2 and the detection of PG [48]. Moreover, it has been demonstrated that palmitoylation of NOD1 and NOD2 is essential for their activation and localisation to the plasma or endosomal membrane during detection of PG or bacteria during infection [49]. However, NOD1 and NOD2 palmitoylation during detection of BMV-associated PG has not been shown. 

In order to have their bacterial ligands accessible to intracellular NLRs, BMVs must first enter host cells. A number of studies have demonstrated the ability of BMVs to enter host cells, enabling their cargo to be detected by NLRs, resulting in the induction of downstream inflammatory signalling pathways. BMVs can enter host cells via a range of mechanisms, including clathrin-dependent or caveolin-mediated endocytosis, lipid raft dependent entry and by membrane fusion [10,27,32,50,51,52,53,54,55,56,57]. Additionally, the size of OMVs has been shown to affect the mechanism of host cell entry and may affect the type of cargo delivered to the host cell, and bacterial growth stage can also regulate the cargo composition of BMVs [58,59].

Once intracellular, BMV-derived MAMPs can then be detected by NLRs. It was initially reported that OMVs produced by the mucosal pathogens *H. pylori*, *P. aeruginosa* and *N. gonorrhoeae* entered non-phagocytic epithelial cells in a lipid raft dependent manner, rendering their PG accessible to detection by NOD1. This in turn resulted in the activation of NF-ĸB, the production of IL-8 and the upregulation of human β-defensins (Table 1, Figure 2A) [10]. Furthermore, it was shown that NOD1 was essential for the generation of *H. pylori*-OMV specific antibody responses when OMVs were administered orally to mice, as NOD1 knockout mice were unable to raise an antibody response to *H. pylori* OMVs (Table 1, Figure 2A) [10]. Therefore, BMVs are a mechanism used by bacteria, irrespective of their mode of infection, to deliver their PG-cargo to the cytosol, resulting in the activation of NOD1 and subsequently a proinflammatory response (Table 1, Figure 2A) [10]. It was subsequently shown that BMVs produced by a range of pathogens could activate NOD1, for example *H. pylori* and *P. aeruginosa* OMVs were shown to enter host epithelial cells which were detected by NOD1, resulting in the recruitment of RIPK2 to early endosomes and the activation of autophagy in the host cell (Table 1, Figure 2A) [31]. Interestingly, autophagosome formation was not induced in immune cells in response to stimulation with *H. pylori* OMVs, which may be due to differences in the mechanism of OMV entry into phagocytic cells [31]. Furthermore, OMVs produced by *Vibrio cholerae* were shown to activate both NOD1 and NOD2 in epithelial and myeloid cells. This was dependent on the quorum-sensing regulator HapR, which acts as a switch for virulence gene expression in *V. cholerae*, whereby OMVs produced by *hapR* knockout strains were significantly decreased in their abilities to induce NOD1 and NOD2 activation in vitro [29]. NOD1 and NOD2 activation by *V. cholerae* OMVs also induced the activation of NF-ĸB and IL-8 in HEK293T cells, and IL-8 production in HeLa and THP1 monocytes [29]. Similarly, *V. cholerae* OMVs were also shown to induce NOD1 activation in HEK293 epithelial cells resulting in IL-8 production and an increase in NOD1 expression. Additionally, dendritic cells obtained from peripheral blood mononuclear cells (PBMCs) which were cocultured with OMV-treated epithelial cells had an increase in the expression of surface markers, such as cluster of differentiation 80 (CD80) and CD83 by dendritic cells, which may contribute to specific OMV-induced adaptive responses [30]. Periodontal pathogens have also been shown to produce OMVs which activate NOD1 and NOD2 dependent responses. For example, *Aggregatibacter actinomycetemcomitans* is a periodontal pathogen that produces OMVs which are internalised by host epithelial cells and monocytes. These OMVs activate NOD1 and NOD2 in HEK293T and THP1-Blue cells, resulting in NF-ĸB activation [32]. However, not all periodontal pathogens are able to activate NODs, as OMVs produced by the periodontal pathogen *P. gingivalis* could activate both NOD1 and NOD2 leading to NF-ĸB production, whereas OMVs produced by *Treponema denticola* and *Tannerella forsythia* could not mediate NOD1 or NOD2 activation [26]. Collectively, these studies highlight that a number of pathogens produce BMVs that are capable of delivering PG to the cytosol of host cells, where it is then detected by NOD1 and NOD2, resulting in the activation of a proinflammatory response. 

### 3.1. Autophagy Induced by Detection of BMVs by NLRs

Once BMVs enter host cells, the host is able to degrade them via the host cellular degradation pathway of autophagy. NOD1 has a key role in mediating the induction of autophagy in epithelial cells in response to bacterial pathogens [68], and similarly, OMVs are able to induce autophagy in epithelial cells in a NOD1-dependent manner [31]. It was reported that once intracellular, *H. pylori* OMVs induced the formation of autophagosomes in epithelial cells in a NOD1-dependent manner, which subsequently resulted in their degradation via autophagy (Figure 2A) [31]. This finding suggests that once OMVs are internalised, they can be degraded via autophagy to facilitate their clearance from the host. In contrast, NOD1 and NOD2 knockout HCT116 human colon cells treated with *Salmonella* OMVs activated AMP-activated protein kinase (AMPK), which also led to the induction of autophagy, suggesting that pathogen derived OMVs can induce autophagy in host cells by complimentary mechanisms [69]. 

BMVs have also been shown to inhibit the induction of autophagy, as MVs produced by the Gram-positive pathogen *Listeria monocytogenes* were able to inhibit autophagy in host cells, thus contributing to bacterial survival within the host [70]. To this end, it is evident that pathogens may produce BMVs which can induce or inhibit autophagy within host cells to drive pathogenesis. Collectively, these studies reveal that BMVs can promote the induction of autophagy, via NOD1-dependent and independent mechanisms, to promote their clearance in addition to impairing the induction of autophagy to facilitate bacterial survival in the host. These findings highlight the multiple mechanisms whereby bacteria can use BMVs to promote pathogenesis and mediate bacterial survival in the host in a tailored manner as required by the parent bacterium. 

### 3.2. Detection of Commensal Derived BMVs by NODs

BMVs produced by commensal and probiotic bacteria have been demonstrated to prime the immune system and contribute to gut homeostasis in a number of ways. OMVs produced by the commensal *B. fragilis* have been demonstrated to mediate immunity by promoting the production of anti-inflammatory cytokines, such as IL-10 by CD4^+^ T cells, in addition to protecting animals against colitis [71]. Furthermore, OMVs produced by commensal and probiotic strains of *E. coli* were shown to induce the production of pro- and anti-inflammatory cytokines by human PBMCs, which was hypothesised to be a result of NOD signalling [72]. This study demonstrated the release of soluble mediators by OMV-stimulated Caco-2 cell monolayers, which could then interact with PMBCs. Furthermore, OMV-mediated activation of NOD1 in epithelial cells and the subsequent release of cytokines and chemokines may be a mechanism of communication and modulation of responses from underlying immune cells. Cytokines released by Caco-2 cell monolayers which were exposed to commensal and probiotic *E. coli* OMVs could activate dendritic cells (DCs) [73]. However, indirect activation of DCs through Caco-2 monolayers triggered the secretion of pro-inflammatory cytokines at a lower level than direct activation of DCs by OMVs [73]. Overall, these studies demonstrate the vast contribution of microbiota derived OMVs in modulation of both epithelial cells, and subsequent activation of immune cells which ultimately regulate a number of homeostatic functions in the gut.

NLRs are known to make a significant contribution to gut homeostasis and immune modulation, whereby dysregulation or inappropriate activation of NLRs can lead to inflammatory disease [35,74]. Furthermore, perturbation of NOD2 expression may contribute to IBD whereby host cells are not able to effectively clear bacteria. Recent studies have demonstrated the contribution of BMVs produced by commensal and probiotic bacteria to induce the production of anti-inflammatory cytokines by immune cells to ultimately promote gut homeostasis and protect against inflammatory diseases in an NLR-dependent manner. For example, OMVs produced by *B. fragilis* were identified to induce the production of the anti-inflammatory cytokine IL-10 by regulatory T cells (Treg) in a NOD2 and ATG16L1 (autophagy related 16 like 1) dependent manner as NOD2 knockout bone-marrow derived dendritic cells (BMDCs) were found to have reduced anti-inflammatory IL-10 production when treated with OMVs produced by the commensal bacterium *B. fragilis* (Table 1) [67]. This study highlights the contribution of NOD2 and ATG16L1 to the production of anti-inflammatory responses mediated by commensal OMVs and has significant implications for the NLR field due to the association of these host proteins in IBD [67].

Furthermore, BMVs produced by commensal bacteria may promote gut homeostasis as a result of activation of NOD1 and NOD2 in epithelial cells. For example, epithelial cells can detect OMVs produced by commensal and probiotic *E. coli* strains ECOR12 and Nissle 1917, respectively, in a NOD1-dependent manner to ultimately modulate cytokine responses [33]. Specifically, commensal and probiotic *E. coli* OMVs could be internalised by epithelial cells via clathrin-mediated endocytosis [75], resulting in the recruitment of NOD1 to early endosomes. Furthermore, NOD1 activation by *E. coli* OMVs in Caco-2 cells resulted in the production of IL-6 and IL-8, which was also found to be dependent on the recruitment of the adaptor molecule RIPK2 [33]. Overall, it was shown that commensal and probiotic *E. coli* derived OMVs could activate NOD1 but not NOD2, resulting in the activation of NF-ĸB and the production of IL-6 and IL-8. Collectively, these studies highlight that detection of BMVs by NOD1 and NOD2 is complex and may vary between commensal and pathogenic organisms. Further elucidating the mechanisms whereby BMVs produced by pathogenic and commensal bacteria can modulate NLR-mediated responses to promote contrasting outcomes forms the basis of future research endeavours to provide insights into how bacteria harness BMVs to modulate immunity. 

## 4. Inflammasome Activation by BMVs

In addition to NOD1 and NOD2, several NLRs have also been identified as key microbial sensors within the innate immune system. More recently described NLRs, such as NLRP3, NLRC4 and NLRP1, can form inflammasomes that are important for the activation of proinflammatory caspases such as caspase-1, which subsequently leads to the maturation of IL-1β and IL-18 [36]. Inflammasomes are signalling complexes which, when activated, lead to pyroptosis and the release of the proinflammatory cytokines IL-1β and IL-18 from host cells [76,77]. However, in most cells, an activation signal (signal 1 or priming) by PRRs is needed to drive expression of the inflammasome components for full functionality upon its activation by the pathogen (signal 2) [78]. This is defined as canonical inflammasome activation, whereas the activation of caspase-11 in mice or caspase-4 and -5 in humans is defined as a non-canonical inflammasome [79]. The latter caspases are activated by binding to bacterial LPS and have been shown to detect OMVs derived from various bacterial species such as *P. aeruginosa, B. pertussis* and *E. coli,* leading to the activation of inflammasomes and the release of proinflammatory cytokines in vitro and in vivo (Figure 2B) [19,60,61,63]. In this way, BMVs that typically contain a range of different MAMPs are able to deliver to host cells both primary and secondary signals required for the activation of inflammasomes and the subsequent production of proinflammatory cytokines (Figure 2B).

The activation of NLRs other than NOD1 and NOD2 and inflammasome activation by BMVs are emerging areas of research in the BMV field, with limited studies reporting their activation to date. OMVs produced by periodontal pathogens *P gingivalis, T. denticola* and *T. forsythia* were reported to activate inflammasome complexes leading to apoptosis-associated speck-like protein (ASC) speck formation, a hallmark of inflammasome activation, and the production of IL-1β by THP-1 cells (Table 1) [60]. Additionally, *P. aeruginosa* OMVs were also reported to induced speck formation, IL-1β and caspase-1 secretion in host cells, which was dependent on the non-canonical caspase-11 pathway [19]. Specifically, it was shown that macrophages from NLRP3 knockout mice had decreased IL-1β and IL-18 production compared to control macrophages when treated with *P. aeruginosa* OMVs, indicating a role for NLRP3 inflammasome activation in the response to OMVs (Table 1, Figure 2B) [19]. A second study also showed the ability of *P. aeruginosa* OMVs to activate NLRP3 and NLRC4, leading to pyroptosis in murine bone marrow-derived macrophages (BMDMs), and treating BMDMs with OMVs derived from porin-knockout bacteria significantly reduced inflammasome activation and pyroptosis, suggesting a role for porins in this NLR response (Table 1) [64]. 

OMVs produced by *B. pertussis* can also contribute to NLRP3 inflammasome activation in murine BMDMs, as caspase-11, which was activated by cytosolic BMV-associated LPS, together with NLRP3 inflammasome activation resulted in the activation caspase-1 (Table 1, Figure 2B) [63]. Caspase-11 also cleaves gasdermin D (GSDMD), which forms pores in the host cell membrane to subsequently trigger pyroptosis and the release of cytokines (Figure 2B) [63,79]. Stimulation of THP-1 monocytes as well as murine BMDMs with *B. pertussis* OMVs resulted in speck formation and increased IL-1β production in THP-1 cells, and the production of TNFα and IL-6 by murine BMDMs [63]. In addition, *E. coli* OMVs are also able to activate caspase-11 and play a role in NLRP3 inflammasome activation in murine BMDMs (Table 1, Figure 2B) [61]. Furthermore, a second study also showed the contribution of capsase-11 to OMV-mediated pathology, as mice deficient in caspase-11 treated with *E. coli* OMVs had significantly reduced IL-1β and IL-18 cytokine responses, which suggests an important role for caspases and inflammasome activation in response to OMVs [51]. Other Gram-negative pathogens including *N. gonorrhoeae*, uropathogenic *E. coli* and *P. aeruginosa* also produce OMVs that induce mitochondrial apoptosis and NLRP3 activation in murine BMDMs [65]. Collectively, these studies highlight the ability of OMVs produced by a range of Gram-negative pathogens to activate caspase-11 and NLRP3 inflammasome activation in the host, to modulate inflammation and promote pathogenesis. 

To date, there are minimal reports describing the ability of MVs produced by Gram-positive bacteria to mediate inflammasome activation in the host. Recently, it was identified that the Gram-positive bacterium *Staphylococcus aureus* produces MVs that can induce NLRP3 inflammasome activation in macrophages [62,66]. However, further research is required to determine if MVs produced by other Gram-positive bacteria also activate inflammasomes. Understanding the mechanisms whereby BMVs from Gram-positive and Gram-negative bacteria can activate NLRs is important for expanding our knowledge regarding the contributions of BMVs to modulating pathogenesis, inflammation and disease.

## 5. Conclusions

BMVs produced by all bacteria are important activators of NLRs responsible for mediating inflammatory responses that contribute to pathogenesis, along with facilitating immunoregulation within the host. Since the discovery that OMVs produced by Gram-negative bacteria can deliver their PG cargo into the cytoplasm of host cells to activate NOD1 and promote pathogenesis, BMVs produced by pathogens have emerged as nanocarriers that function via a range of mechanisms to activate NLRs and promote disease. However, much still remains to be elucidated regarding the intracellular location where NLRs interact with BMV cargo to mediate their activation, promote inflammation and facilitate inflammasome activation in the host. For example, determining the structure of NLRs, in particular NOD1 and NOD2, will enable us to further understand the atomic detail of how NODs detect BMV-associated cargo and will facilitate the development of NOD antagonists to limit BMV and NOD-mediated inflammation in the host. Due to the implications of OMVs mediating inflammation and promoting pathology during chronic inflammatory conditions mediated by NLRs, such as Crohn’s disease, understanding how OMVs modulate inflammation in the host will ultimately result in the development of novel targets to limit disease. 

Moreover, the recent discovery that BMVs produced by commensal and probiotic bacteria can promote gut homeostasis and modulate NLR-mediated inflammation and immunity is an exciting area of future research. Elucidating the mechanisms whereby commensal OMVs modulate NLR interactions at the mucosal interface will propel our limited understanding of their contribution to facilitating gut homeostasis and their ability to limit pathology in chronic inflammatory conditions such as IBD and Crohn’s disease. Defining the mechanisms whereby BMVs enter host cells, interact with NLRs and regulate their cargo, which is responsible for modulating these NLR-dependent responses, will have enormous implications for developing methods to limit BMV-mediated pathology in addition to harnessing BMVs for future therapeutic use in chronic disease states. 

## Figures and Tables

**Table 1:** Chronological summary of studies demonstrating the detection of pathogen and commensal derived BMVs by NLRs and the downstream consequences.

Pathogen Derived BMV Activation of NLRs				
Bacterial Species Producing BMVs	NLR Activation	Host Cell Type	Outcome of Activation by BMVs	Ref.
*H. pylori* *P. aeruginosa* *N. gonorrhoeae*	NOD1	AGS gastric adenocarcinoma epithelial cells HEK293 epithelial cells	NF-ĸB activation IL-8 ↑ Human-β-defensin 2 ↑ Human-β-defensin 3 ↑	[10]
*V. cholerae* V:5/04	NOD1 NOD2	HEK293T epithelial cells THP-1 myeloid cells	NF-ĸB activation IL-8 ↑	[29]
*V. cholerae* O395	NOD1	HEK293 epithelial cells	IL-8 ↑ (expression and protein level) GM-CSF ↑ (expression and protein level) NOD1 protein↑	[30]
*H. pylori* *P. aeruginosa*	NOD1	HeLa epithelial cells *^#^ Mouse embryonic fibroblasts ^^#^ AGS cells *^#^ Primary human bronchial cells ^#^	Autophagosome induction ↑ ^#^ IL-8 ↑ * CXCL2 ^	[31]
*A. actinomycetemcomitans*	NOD1 NOD2	HEK293T cells THP1-Blue cells with siRNA NOD1 and/or NOD2 KO	NF-ĸB activation	[32]
*P. gingivalis*	NOD1 NOD2	HEK-Blue cell lines	HEK-Blue SEAP reporter system	[26]
*P. gingivalis* * T. denticola *T. forsythia*	NLRP3	Monocytes	NF-ĸB activation TNFα ↑ IL-8 ↑ IL-1β ↑ IL-10 ↑ *	[60]
*E. coli* (K-12 BW25113)	NLRP3	Murine BMDMs	IL-1β ↑ IL-18 ↑	[61]
*P. aeruginosa*	NLRP3	THP-1 monocytes	IL-1β ↑ IL-18 ↑	[19]
*S. aureus*	NLRP3	THP-1 monocytes	IL-1β ↑ IL-18 ↑	[62]
*B. pertussis*	NLRP3	THP-1 monocytes Murine BMDMs	IL-1β ↑ IL-18 ↑	[63]
*P. aeruginosa*	NLRP3 NLRC4	MH-S murine alveolar macrophage cells	IL-1β ↑	[64]
*N. gonorrhoeae* Uropathogenic *E. coli* (CFT073) *P. aeruginosa*	NLRP3	Murine BMDMs	IL-1β ↑	[65]
*S. aureus*	NLRP3	THP-1 monocytes	IL-1β ↑ IL-18 ↑	[66]
**Commensal derived BMV activation of NLRs**				
*B. fragilis*	NOD2	NOD2 KO mouse BMDCs	IL-10 ↓	[67]
*E. coli* (ECOR12, Nissle 1917)	NOD1	Caco-2 epithelial colonic cell line Caco-2 cells with NOD1 siRNA	NF-ĸB activation IL-6 ↑ IL-8 ↑	[33]

^#^^* Cell type- or bacterial species-dependent responses observed within each row.
